# A Web-Based Telemanagement System for Improving Disease Activity and Quality of Life in Patients With Complex Inflammatory Bowel Disease: Pilot Randomized Controlled Trial

**DOI:** 10.2196/11602

**Published:** 2018-11-27

**Authors:** Javier Del Hoyo, Pilar Nos, Raquel Faubel, Diana Muñoz, David Domínguez, Guillermo Bastida, Bernardo Valdivieso, Marisa Correcher, Mariam Aguas

**Affiliations:** 1 Gastroenterology Department La Fe University and Polytechnic Hospital Valencia Spain; 2 CIBEREHD (Networked Biomedical Research Center for Hepatic and Digestive Diseases) Valencia Spain; 3 Health Research Institute La Fe Valencia Spain; 4 Physiotherapy Department University of Valencia Valencia Spain; 5 Joint Research Unit in Biomedical Engineering (eRPSS: IIS La Fe-UPV) Valencia Spain; 6 Connected Health Services Valencia Spain; 7 Home Care and Telemedicine Department La Fe University and Polytechnic Hospital Valencia Spain; 8 Red de Investigación en Servicios de Salud en Enfermedades Crónicas (REDISSEC) Madrid Spain; 9 Systems Department La Fe University and Polytechnic Hospital Valencia Spain

**Keywords:** Crohn disease, e-health, inflammatory bowel disease, information and communication technology, telemedicine, ulcerative colitis

## Abstract

**Background:**

The reported efficacy of telemedicine in patients with inflammatory bowel disease (IBD) is inconsistent among studies, and data for complex IBD are lacking.

**Objective:**

We aimed to evaluate the impact of remote monitoring using a Web system—*Telemonitorización de la Enfermedad de Crohn y Colitis Ulcerosa* or Telemonitoring of Crohn’s Disease and Ulcerative Colitis (TECCU)—as compared to standard care and telephone care on health outcomes and health care in patients with complex IBD.

**Methods:**

We performed a 3-arm randomized controlled trial. Adult patients with IBD who received immunosuppressants and biological agents were recruited from the IBD Unit of a tertiary university hospital. The patients were randomized into groups to receive remote monitoring (G_TECCU), nurse-assisted telephone care (G_NT), or standard care with in-person visits (G_control). All patients completed the study visits at baseline and at 12 and 24 weeks in addition to each type of intervention. The primary outcome was the percentage of patients in remission at 24 weeks. Secondary health outcomes were quality of life, medication adherence, adverse effects, satisfaction, and social activities. Data on the number of outpatient visits and telephone calls, emergency visits, hospitalizations, IBD-related surgeries, and corticosteroid courses were also collected.

**Results:**

A total of 63 patients were selected (21 patients in each group). During the study, 90.5% (19/21) of patients in G_control, 95.2% (20/21) in G_NT, and 85.7% (18/21) in G_TECCU were compliant to the intervention. After 24 weeks, the percentage of patients in remission was higher in G_TECCU (17/21, 81%) than in G_NT (14/21, 66.7%) and G_control (15/21, 71.4%). A higher improvement in disease activity was observed in G_TECCU than in G_control in terms of the Harvey-Bradshaw/Mayo (odds ratio=0.12, 95% CI=0.003-2.162, *P*=.19) and Harvey-Bradshaw/Walmsley (odds ratio=0.11, 95% CI=0.004-1.55, *P*=.13) indexes. Improvement in disease activity was associated with a larger reduction in fecal calprotectin values in G_TECCU compared to G_control (estimated intervention effect: odds ratio=–0.90; 95% CI=–1.96 to 0.16, *P*=.11). All completers adhered to treatment in G_TECCU. In addition, the quality of life, social activities, and satisfaction improved in all 3 groups. Although the number of outpatient visits and telephone calls was lower in G_TECCU than in G_NT and G_control, the safety profile was similar in all 3 groups.

**Conclusions:**

This pilot clinical trial suggests that the TECCU Web-based system is a safe strategy for improving health outcomes in patients with complex IBD and reducing the use of health care resources.

**Trial Registration:**

ClinicalTrials.gov NCT02943538; https://clinicaltrials.gov/ct2/show/NCT02943538 (Archived by WebCite at http://www.webcitation.org/746CRRtDN).

## Introduction

Inflammatory bowel disease (IBD), comprising ulcerative colitis (UC) and Crohn disease (CD), is a chronic relapsing disorder characterized by inflammation of the gastrointestinal tract. Its natural progression includes flares and periods of remission, and IBD requires continuous and personalized follow-up to ensure long-term remission. Patients with IBD use health care resources significantly more often than patients with other conditions [1]. Importantly, 30%-45% of adult patients with IBD are nonadherent to treatment [2], which increases the probability of relapse by 4-fold and consequently increases the health care costs [3]. The high percentage of nonadherence is associated with behavioral and psychological factors and the physician-patient relationship [2]. In addition, IBD is related to high levels of school absenteeism and work disability [4], interference with social activities, and impairment of health-related quality of life (HRQoL) [5]. Therefore, IBD has a significant medical, social, and financial impact.

To address these difficulties, telemedicine uses information and communication technologies (ICTs) to provide health care services remotely. Despite the heterogeneous nature and progress of IBD, ICTs allow tailored follow-up with better communication between physicians and patients [6-8] and provide educational resources that promote patient empowerment [6] and optimize treatment [9,10]. ICTs were initially used to support the management of other chronic diseases such as congestive heart failure [11], diabetes mellitus [12], and chronic obstructive pulmonary disease [13] and showed excellent acceptance by patients and improvement in HRQoL. Owing to these positive results, telemedicine systems have been evaluated in patients with IBD [14,15].

A ground-breaking evaluation of telemedicine in patients with IBD by Cross et al. [16], who designed the Home Automated Telemanagement system, was acceptable, increased patients’ knowledge about their disease, and facilitated greater self-control of IBD symptoms [17]. However, in a randomized controlled trial of 47 patients with UC in remission or with mild UC activity, no significant differences were noted in disease activity, HRQoL, or medication adherence between patients who followed the telemedicine system and those who received usual care at 12 months [18]. These results were partially explained by a higher discontinuation rate in the Home Automated Telemanagement group due to the platform design. To avoid similar problems, the authors subsequently designed a mobile phone-based Web program to determine the impact of telemedicine on health outcomes [19]. Web programs and mHealth interventions are increasingly used to provide information and health care at a distance. Unlike other telehealth systems, Web-based apps do not require home installation and are less expensive. Web-based systems adapted to IBD are safe and feasible for adults and adolescents [20-22], improve medication adherence, and reduce the duration of relapses [6]. A recent multicenter, randomized controlled trial including more than 900 patients with different subtypes of IBD showed that compared with standard care, empowerment through these systems reduces outpatient visits and hospital admissions, with potential cost savings [8]. Web-based systems are currently the most useful platforms, and their application allows development of distance care projects aimed at providing access to health care in remote areas [23,24]. These models promote collaboration and knowledge exchange between specialists, potentially reducing variability in clinical practice, and could modify the future structure of health systems if they prove to be cost-effective. However, the effect of telemedicine systems on disease outcomes is inconsistent and varies across studies according to the population and health care system in which they are applied [6,8,18,20,22]. Moreover, no specific studies have thus far focused on the impact of telemedicine in patients with complex IBD.

Our research group designed a Web-based telemanagement system known as *Telemonitorización de la Enfermedad de Crohn y Colitis Ulcerosa* or Telemonitoring of Crohn’s Disease and Ulcerative Colitis (TECCU) for remote monitoring of patients with moderate-to-severe IBD activity or who initiate treatment with corticosteroids, immunosuppressants, and biological agents. We performed a randomized controlled trial to assess the safety of care provided through this system and its impact on disease activity, HRQoL, medication adherence, satisfaction, work productivity, social activities, and use of health care resources and compared these outcomes with those of nurse-assisted telephone care and standard face-to-face visits.

## Methods

### Study Design

A 3-arm, parallel-group, randomized controlled trial was performed to compare the impact of the Web-based telemanagement system TECCU, nurse-assisted telephone care, and standard face-to-face visits on health outcomes and outpatient visits in patients with complex IBD. Neither the patients nor the researchers were masked to the intervention, but the results were analyzed by an independent statistician who was blinded to group identification.

#### Ethical Considerations

The study protocol was reviewed and approved by the local independent ethics committee of La Fe University and Polytechnic Hospital, Valencia, Spain; the regional independent ethics committee (Comité Ético Autonómico de Estudios Clínicos de Medicamentos y Productos Sanitarios de la Comunitat Valenciana); and the Spanish Agency of Medicines and Medical Devices (Agencia Española de Medicamentos y Productos Sanitarios). According to the physicians involved in the study, the risks did not outweigh the potential benefits, and each participant provided informed consent without coercion before inclusion in the study. The trial is registered at ClinicalTrials.gov with the identifier NCT02943538.

### Patient Selection

Patients were recruited from the IBD Unit of a tertiary university hospital. All participants were diagnosed with IBD at least 6 months previously and according to the internationally accepted criteria [25,26].

#### Inclusion and Exclusion Criteria

The inclusion criteria were age ≥ 18 years; initiation of therapy with corticosteroids, immunosuppressants, and biological agents due to disease activity; and provision of written informed consent to participate in the study. The exclusion criteria were inability to speak and read Spanish; inability to manage a mobile phone or tablet or the Internet or not having a telephone line; participation in other clinical trials during the inclusion period; uncontrolled medical or psychiatric disease; presence of ileorectal or ileal pouch-anal anastomosis; receipt of definitive ileostomy; perianal disease; and pregnancy.

#### Recruitment

This project was funded in 2012, and the platform was installed and configured in 2013-2014. Enrollment was started in October 2014 and ended in June 2016. The follow-up ended in December 2016. Patients were included consecutively from the Outpatient Clinic of the IBD Unit or the Gastroenterology Department if they were admitted for a flareup of IBD. During the visit, the inclusion and exclusion criteria were verified, and the patient was informed about the study through the patient-information sheet. If the patient agreed to participate, he or she signed the informed consent document.

#### Randomization

Eligible patients were randomized to 1 of the 3 groups to receive remote monitoring (G_TECCU), nurse-assisted telephone care (G_NT), or standard care with in-person visits (G_control) in a 1:1:1 ratio by using a block randomization method through a Web-based tool [27] in order to generate a random-allocation sequence and ensure allocation concealment. Once a number was assigned, it could not be reassigned, and members of the research team who were in contact with patients did not have access to the randomization tables or lists.

### Study Outcomes

Participants completed study visits at the baseline, 12 weeks, and 24 weeks, in addition to routine visits scheduled for their clinical care. The variables measured at baseline were sociodemographic variables, disease profile and activity, HRQoL, adverse events, medication adherence, and patient satisfaction.

#### Primary Outcome

The primary objective of the study was to determine the percentage of patients in clinical remission at 24 weeks. Remission was evaluated using the modified Harvey-Bradshaw index (HBI) for patients with CD [28]. For patients with UC, we used the Simple Clinical Colitis Activity Index (SCCAI, also known as the Walmsley index) [29] for remote checkups together with the partial Mayo score for face-to-face visits [30].

Patients with CD and an HBI < 5 were considered to be in clinical remission, whereas patients with scores of 5-7, 8-16, or >16 were considered to have mild, moderate, or severe activity, respectively [28]. For remote checkups in patients with UC, clinical remission was defined as a Walmsley score ≤ 2, whereas mild-to-moderate and severe activities were defined as scores of 3-5 and >5, respectively [31]. In the face-to-face visits, clinical remission was defined as a partial Mayo score ≤ 2 and no individual Mayo subscore > 1; scores of 2-5, 6-8, and 9 were defined as mild, moderate, and severe disease activity, respectively [30].

Laboratory parameters were measured at baseline and each subsequent visit according to individual patient’s schedule and included a complete blood analysis with nutritional profile and C-reactive protein level (mg/L). Fecal calprotectin (FC, µg/g) was assessed at baseline, 12 weeks, and 24 weeks after inclusion. Changes in the medication were made on the basis of the results obtained, in association with clinical activity indexes, and were prescribed according to specific intervention plans adapted to the severity of each alert.

#### Secondary Outcomes

The HRQoL of patients at inclusion and at week 24 was evaluated using the specific questionnaire Inflammatory Bowel Disease Questionnaire 9 (IBDQ-9) and the generic questionnaire EuroQol-5D (EQ-5D). IBDQ-9 is a validated questionnaire comprising 9 items distributed in 4 dimensions: bowel symptoms, systemic symptoms, emotional function, and social function. It correlated very well with the Spanish version of the 36-item IBDQ [32]. Each item is scored on a 7-point Likert scale, with an overall score ranging from 7 (lowest QoL) to 63 (highest QoL) points, and calculated over 100 points. EQ-5D is a generic questionnaire that has been used for patients with various chronic diseases such as IBD and was previously validated in Spain [33]. This instrument provides a global value for HRQoL, with 5 questions related to 5 dimensions: mobility, selfcare, usual activities, pain or discomfort, and anxiety or depression. Additionally, a visual analog scale (VAS) was used. Each question was scored with 1, 2, or 3 points, depending on whether impairment in the dimension assessed was nonexistent, moderate, or extreme, respectively; subsequently, the results were converted for each dimension according to the specific coefficients calculated for the Spanish population [34]. The maximum score of 1 represents the best health status, a score of 0 represents a health state considered equivalent to death, and negative index scores represent health states considered worse than death. The VAS rates overall health between 0 (poorest imaginable health) and 100 (best imaginable health).

We assessed the impact of disease on work productivity and activities of daily living using the Work Productivity and Activity Impairment (WPAI) questionnaire [35], which patients completed at baseline and week 24. The questionnaire comprises 6 questions on the effect of the disease on work and activities of daily living during the previous 7 days. A higher score on the questionnaire indicates a more pronounced effect on work and daily activities. The Spanish version has been validated and is reproducible in patients with CD.

Medication adherence was evaluated using the Morisky-Green index [36], which has been used to evaluate adherence in patients with IBD in clinical trials [18]. We considered adherence to be adequate when the patient answered all questions correctly and inadequate if any answer was associated with nonadherence.

We recorded the number of outpatient visits and telephone consultations, as registered in the NOMHADCHRONIC app (version V2.RC6; Connected Health Services SL, Valencia, Spain) for patients in G_TECCU and in the Orion Clinic information system (as part of daily clinical practice at our hospital) for all 3 groups during the study. Patients who adhered to >80% of checkups in the study protocol were considered to be compliant.

The safety of each intervention was assessed by measuring the number of visits to the emergency department, hospitalizations, IBD-related surgeries, corticosteroid courses, and adverse effects to medication. Finally, patient satisfaction with the care received was evaluated at 24 weeks by using an adapted version of the Client Satisfaction Questionnaire, which comprises 6 questions (measured on a scale of 0-10) on the quality, usefulness, and viability of the system of care applied for each case [37].

### Setting and Interventions

This pilot trial was developed at the tertiary referral center La Fe University and Polytechnic Hospital, Valencia, Spain. The hospital serves more than 1500 patients with IBD, has 2 specialist IBD nurses, and provides an email and telephone consultation structure for IBD patients to contact the hospital.

The trial had 3 arms and compared remote monitoring through a Web-based telemanagement system, nursing care by telephone, and usual care provided in our IBD Unit (Outpatient Clinic). All patients completed the study visits at baseline and 12 and 24 weeks, in addition to routine visits to the IBD clinic, telephone consultations, or Web telemonitoring based on group assignment at randomization. Disease activity, HRQoL, adverse effects, adherence, and use of health care resources were measured at baseline and during the 24-week follow-up.

To adequately control disease activity and adverse effects of immunosuppressants, patients treated with these drugs alone or in combination with biological agents were monitored every 1-2 weeks during the first month, every 2-4 weeks between months 1 to 3, and every 4 weeks from month 3 until the end of follow-up. Patients treated with biological agents alone were monitored every 2-4 weeks during the entire follow-up period. Patients from all 3 arms who took the same type of drug underwent these follow-up schedules; the schedules only differed in terms of the monitoring: patients in G_TECCU were monitored via the NOMHADCHRONIC app, those in G_NT were monitored via a telephone line managed by nursing staff from the IBD Unit, and those in G_control were monitored by the usual face-to-face visits combined with telephone calls made by physicians according to standard clinical practice. Moreover, additional clinical visits were performed, if required, during patient evolution in any of the 3 arms.

#### Telemonitoring Apps

In G_TECCU, follow-up and monitoring were performed telematically using the integrated platform for management of chronically ill patients (NOMHADCHRONIC app), which was designed to meet the specific needs of this group. The NOMHADCHRONIC platform is an innovative technological system that was designed to boost the rollout of services for the management of chronically ill patients. The telemanagement care system was developed in collaboration with patients, the La Fe telemedicine Unit, and *Tecnologías para la Salud y el Bienestar* SA. The protocol of the Web-based telemanagement system (TECCU) has been described elsewhere [27]. TECCU is a secure webpage with an HTTP app for mobile phones, tablets, and computers. Patients connected to the platform via the Internet using a computer or an app on a mobile phone or tablet had to self-complete questionnaires. In addition, they received advice, reminders, educational material about their disease, and information on prevention. This information was received by the case managers and filtered using an intelligent prioritization system with generation of alerts and push notifications according to an integrated intervention protocol.

Patients monitored via the TECCU used NOMHADhome [38]. The resources of the NOMHADhome platform were also available on the NOMHAD mobile app, which patients could download onto their mobile phones. After inclusion, these patients automatically received an email with a personal code that allowed them to access the platform. Each patient profile contained the following information: contact information, active IBD medications, testing schedule (blood and stool tests), log of disease activity, medication use, body weight, vital signs, HRQoL, alerts and action plans, progress of inflammatory activity and vital signs in the form of graphs, electronic messaging to the study nurse coordinator and health care provider, and educational tips.

**Figure 1:**
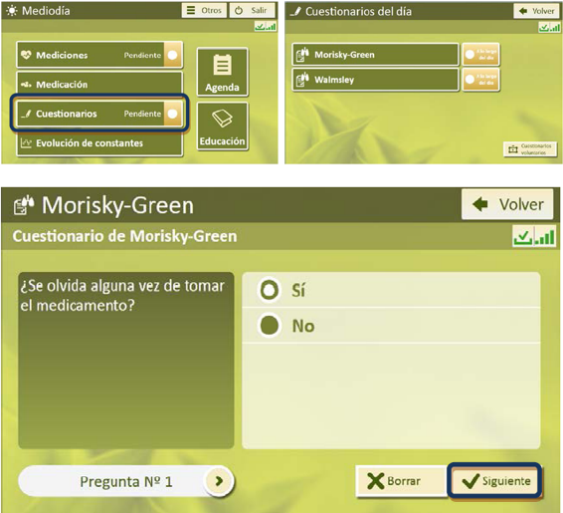
Homepage of the NOMHADhome platform for access to questionnaires (patient version).

**Figure 2:**
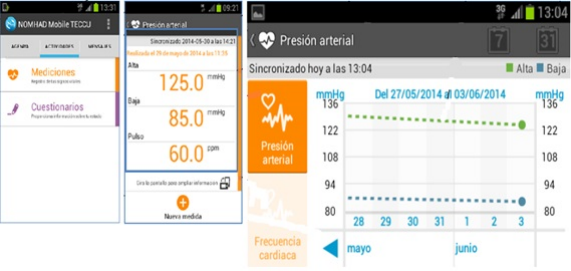
Home page of the NOMHADmobile platform for access to vital signs (patient version).

Patients in G_TECCU had to answer several simple questions related to their IBD symptoms and possible adverse effects since the last evaluation via text messaging. In the main menu of the platform, patients accessed the questionnaires by clicking on specific icons designed for this purpose and could answer the multiple choice and yes or no questions needed to complete these items (Figures 1 and 2). We established individualized alerts and action plans based on the answers to questions about the activity index, adverse effects, and blood biochemistry results. A scale of values was assigned for each alert depending on the severity (green, yellow, orange, and red zone).

After receiving an alert, the specialized medical staff, in collaboration with the nurses, used the general recommendations of the action plans to guide medication adjustments in biological therapy, immunomodulators, corticosteroids, and salicilates. These treatment changes were performed with the support of the platform messaging system in combination with telephone calls or in-person visits when it was necessary to train patients for the administration of medications such as subcutaneous biological agents. Once the disease was in remission again (green zone), the patient had to continue with the initially programmed follow-up. All patients had access to tools that informed them about their disease. In G_TECCU, information was available on the NOMHADhome platform itself; in G_control and G_NT, patients received written documents with the same information.

#### Usual Care Provided in the Inflammatory Bowel Disease Unit

The G_control patients received the normal care provided in the IBD Unit (Outpatient Clinic) for patients with moderately to highly complex IBD, based on national and European clinical guidelines [25,26,39]. Treatment was adjusted according to the evolution of disease activity and medication adherence, which was measured using specific indexes and biological markers used to report the study outcomes during office visits or telephone calls. In addition, to measure the time in remission, clinical activity of patients was self-recorded in a diary on paper at home weekly during the first 12 weeks and every 2 weeks subsequently, until the end of the follow-up.

This care was complemented by ad hoc hospital care in case of flareups or if the patient’s health deteriorated for any reason. Ad hoc intensive care was maintained until the patient’s condition stabilized, at which point he or she returned to follow-up based on standard care in the Unit. For comparability among the 3 groups, we provided information on all available on-paper educational materials about IBD for remotely monitored patients as well as information on prevention and written action plans in case of flareups. Questionnaires on HRQoL, satisfaction, and work productivity were recorded at baseline and at the end of the study.

#### Nursing Care by Telephone

The G_NT patients were asked about their health through telephone calls by the nursing staff in the IBD Unit. We performed telephone assessment periodically by using structured interviews to evaluate health status, and clinical activity was self-recorded at home, as described for the G_control patients. The interventions depended on the results of the interview and changes in the medication or follow-up schedule established by nurses with the support of medical staff, according to the alerts and action plans designed in the intervention protocol [27]. Furthermore, we provided these patients with all educational elements made available to the other 2 groups.

### Notification of Adverse Effects

TECCU is a minimum-risk system. The characteristics of the interventions in the present study are designed such that patients do not experience direct adverse effects. The interventions were proposed to control and achieve long remission periods in patients with IBD and were not expected to cause lesions or damage to the patients’ health, since we did not test a new experimental drug. We evaluated a Web-based telemonitoring system designed to improve communication between patients and health care providers, with a proactive remote follow-up method, allowing changes in the patients’ treatment and follow-up schedule when flareups are detected or when the flareups increase in severity as per the alert system. In addition, we incorporated educational elements in G_TECCU and the other 2 groups to make them more comparable.

With respect to the platform, a risk-minimization study was performed to ensure accomplishment of ethical norms and that the appropriate ethics committees approved the study. We also recorded all adverse events from the time the patient provided consent to participate in the study until 28 days after study completion.

### Power Calculation

The most-efficient means of determining differences between the 3 groups was by contrasting differences in activity indexes for CD and UC. Considering the differences in the scales, the analysis was stratified by performing a comparison of patients with UC and another comparison of patients with CD. The sample size was calculated by estimating the size required to detect a difference of 3 points in the HBI; considering an SD of 4, a power of 80%, and an alpha significance level of .05, a total of 30 patients with CD (10 per arm) was required. In addition, for a 2-point difference in the Mayo index, an SD of 2.5, a power of 80%, and an alpha significance level of .05, a total of 30 patients with UC (10 per arm) was required. Therefore, the overall sample size was 60 patients (20 per arm). We also stratified patients globally (CD and UC) by comparing patients in remission or with inflammatory activity, irrespective of disease severity (mild, moderate, or severe).

### Statistical Analysis

First, we described the characteristics of patients in the test and control groups by applying appropriate estimators (means, medians, or proportions) according to the type of variables and evaluated possible differences between the groups in the main and secondary outcomes by using regression models to assess differences in these measures. We evaluated the effects of the different treatment groups on the probability of remission using mixed-effects logistic regression models including an interaction between week and treatment groups. Individuals were included in the model as a random intercept to correct for the nonindependent data. Differences in slopes for weeks 12 and 24 were evaluated by assessing the interaction between week and treatment groups. *P* values were estimated using the Satterthwaite approximation for degrees of freedom. We used mixed-effects ordinal logistic regression models to evaluate progress according to the IBDQ-9 and EQ-5D scores in the 3 study groups between weeks 0 and 24. However, owing to convergence problems in assessing medication adherence (because of the reduced sample size and the ordinal character of the Morisky-Green test), Bayesian techniques were applied in the ordinal logistic regression model. Differences in the percentage of missed work hours, work impairment, and social impairment were analyzed using mixed-effects linear regression and mixed-effects beta regression models (% work hours missed). Profile likelihood 95% confidence intervals were calculated for all estimations, and *P*<.05 was considered statistically significant. A likelihood-based analysis for all patients included was performed using R (version 3.5.1) with the lme4 (version 1.1-17), lmerTest (version 3.0-1), brms (version 2.2.0), clickR (version 0.4.04), ordinal (version 2018.4-19), and glmmADMB (0.8.3.3) packages [40].

## Results

### Study Sample

**Figure 3:**
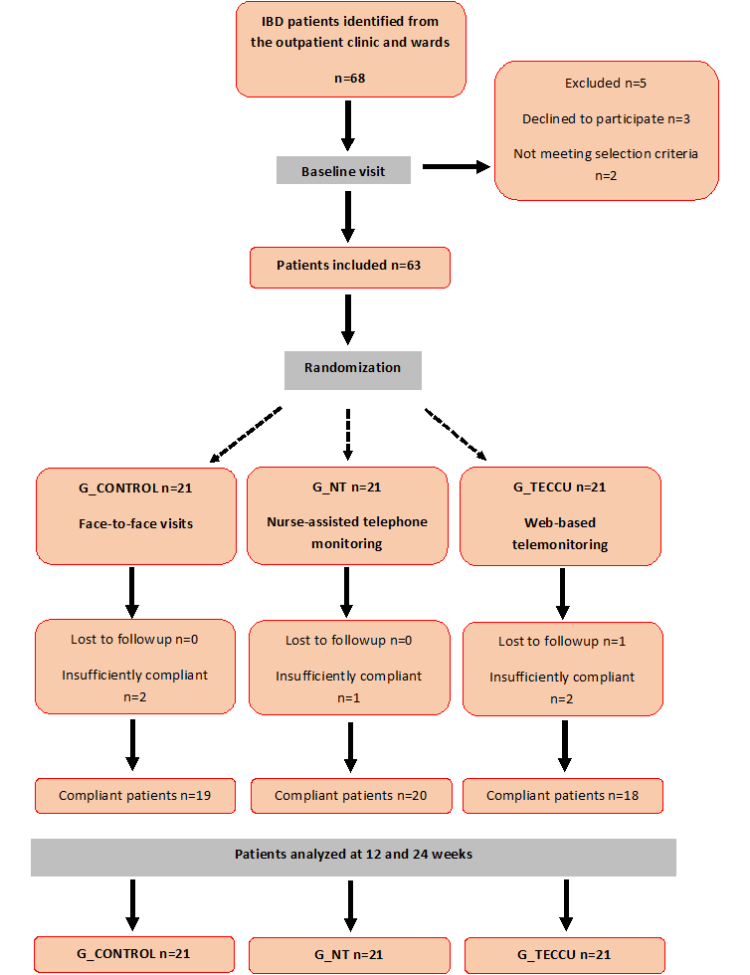
Flowchart of study participants. G_CONTROL: group receiving standard care with in-person visits; G_NT: group receiving nurse-assisted telephone care; G_TECCU: group receiving remote monitoring; IBD: inflammatory bowel disease.

A total of 68 patients with complex IBD were invited to participate in this study between October 2014 and June 2016, of which 3 (4.4%) declined to participate owing to inaccessibility to the Internet at home and 2 (2.9%) did not meet the inclusion criteria. The remaining 63 eligible patients provided informed consent and were randomly assigned to the 3 groups (21 patients in each group, Figure 3). During the study period, all patients except 1 patient in G_TECCU (95.2%) continued to use the Web-based telemanagement system and 2 other patients did not respond to >80% of checkups. The remaining 18 patients (85.7%) in G_TECCU showed good adherence to the study protocol as compared to 19 patients (90.5%) in G_control and 20 patients (95.2%) in G_NT. We did not observe any differences between the completers and patients who did not adhere to the study protocol. The baseline characteristics of the 3 study groups are shown in Table 1.

### Disease Activity

**Figure 4:**
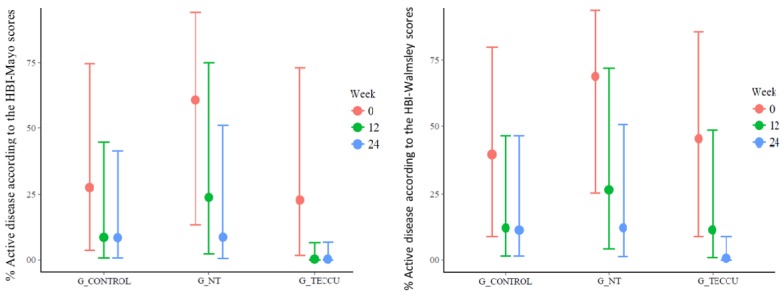
Evolution of disease activity over the study period in the 3 groups. G_CONTROL: group receiving standard care with in-person visits; G_NT: group receiving nurse-assisted telephone care; G_TECCU: group receiving remote monitoring; HBI: Harvey-Bradshaw index.

According to the HBI and the partial Mayo scores, 47.6% (10/21), 38.1% (8/21), and 57.1% (12/21) of patients were in clinical remission at baseline in G_TECCU, G_NT, and G_control, respectively. The baseline percentage of remission according to the Walmsley score for UC was the same for each group and improved progressively at 12 and 24 weeks in all 3 groups. This percentage was higher in G_TECCU, even after considering dropouts, with 81% of patients (17/21) inactive after 24 weeks compared with 66.7% (14/21) in G_NT and 71.4% (15/21) in G_control (using both Mayo and Walmsley scores for evaluation of UC); therefore, the percentage of patients in remission increased by 33.4% in G_TECCU, 28.6% in G_NT, and 14.3% in G_control (Figure 4). In the intention-to-treat analysis, a higher probability of remission was observed in G_TECCU than in G_control at 12 weeks (odds ratio (OR)=0.12, 95% CI=0.003-2.103, *P*=.18) and 24 weeks (OR=0.12, 95% CI=0.003-2.162, *P*=.19) using the HBI-Mayo scores. With the HBI-Walmsley scores, no differences were observed between G_TECCU and G_control at 12 weeks (OR=0.92, 95% CI=0.08-9.71, *P*=.94), but the probability of remission was higher in G_TECCU at 24 weeks (OR=0.11, 95% CI=0.004-1.55, *P*=.13). The median time in remission was 17.9 weeks (interquartile range, 12-24 weeks) in G_TECCU compared to 17.3 weeks (interquartile range, 10-24 weeks) in G_NT and 14.3 weeks (interquartile range, 8-24 weeks) in G_control.

**Figure 5:**
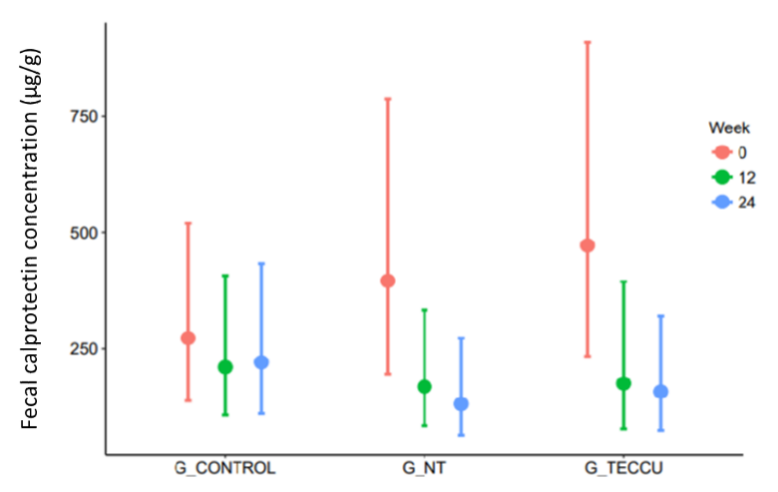
Evolution of the median fecal calprotectin levels over the study period in the 3 groups. G_CONTROL: group receiving standard care with in-person visits; G_NT: group receiving nurse-assisted telephone care; G_TECCU: group receiving remote monitoring.

Disease activity was evaluated on the basis of FC levels throughout the study. At 24 weeks, the median FC level for clinical activity improved progressively from a baseline value of 490 µg/g to 137 µg/g in G_TECCU and from 526 µg/g to 115.5 µg/g in G_NT; however, this reduction was smaller in G_control, from 330 µg/g to 230 µg/g. The improvement in FC levels was larger in G_TECCU than in G_control, with an estimated intervention effect of –0.76 (95% CI=–1.85 to 0.336, *P*=.18) in the reduction of FC values at 12 weeks and –0.90 (95% CI=–1.96 to 0.16, *P*=.11) at 24 weeks. Similarly, a larger improvement was noted in G_NT than in G_control at the same time points, with an estimated intervention effect of –0.61 (95% CI=–1.62 to 0.39, *P*=.25) at 12 weeks and –0.91 (95% CI=–1.96 to 0.15, *P*=.10) at 24 weeks (Figure 5).

**Table 1:** Baseline characteristics of patients.

Characteristics		Control group (n=21)	Telephone group (n=21)	TECCU^a^ group (n=21)
Median age, years (range)		39.31 (22-61)	40.91 (24-60)	41.32 (19-66)
**Sex, n (%)**				
	Men	12 (57.1%)	12 (57.1%)	9 (42.9%)
	Women	9 (42.9%)	9 (42.9%)	12 (57.1%)
**Education, n (%)**				
	Primary	4/21 (19%)	4/21 (19%)	5/21 (23.8%)
	Secondary	9/21 (42.9%)	6/21 (28.6%)	6/21 (28.6%)
	University	8/21 (38.1%)	11/21 (52.4%)	10/21 (47.6%)
**Disease profile**				
	Crohn disease, n (%)	14/21 (66.7%)	13/21 (61.9%)	13/21 (61.9%)
	Ulcerative colitis, n (%)	7/21 (33.3%)	8/21 (38.1%)	8/21 (38.1%)
Median time since diagnosis, months (range)		123.32 (6-427)	108.27 (7-452)	146.72 (7-424)
**Treatment (%)**				
	Immunomodulators	10/21 (47.6%)	10/21 (47.6%)	9/21 (42.9%)
	Biological monotherapy	4/21 (19%)	4/21 (19%)	4/21 (19%)
	Combination therapy	6/21 (28.6%)	5/21 (23.8%)	6/21 (28.6%)
	Corticosteroids	1/21 (4.8%)	2/21 (9.5%)	2/21 (9.5%)
**Clinical Remission, n (%)**		12/21 (57.1%)	8/21 (38.1%)	10/21 (47.6%)
	Crohn disease	10/14 (71.4%)	6/13 (46.2%)	9/13 (69.2%)
	Ulcerative colitis	2/7 (28.6%)	2/8 (25%)	1/8 (12.5%)
Median calprotectin level, μg/g (interquartile range)		330 (103-617)	526 (115-1724)	490 (23-2016)
**Quality of life**				
	Median IBDQ-9^b^ score (interquartile range)	38.50 (33.25-46.75)	37.50 (28.75-46.25)	42.00 (33.75-47.50)
	Median EQ-5D^c^ score (interquartile range)	0.816 (0.754-0.914)	0.825 (0.710-0.914)	0.825 (0.576-0.914)
	Median VAS^d^, % (interquartile range)	60.5% (50%-85%)	62.5% (50%-80%)	60% (40%-90%)
Medication adherence, n (%)		14/21 (66.7%)	7/21 (33,3%)	12/21 (57,1%)
**WPAI^e^**				
	Not working, n (%)	8/21 (38.1%)	7/21 (33.3%)	5/21 (23.8%)
	Percentage of work hours missed, median (interquartile range)	27.5% (0%-52%)	40% (15%-62.5%)	32.5% (7.5%-57.5%)
	Work impairment score, median (interquartile range)	7 (2.75-10)	7 (3-10)	10 (2.25-10)
	Social impairment score, median (interquartile range)	3.5 (1-5.75)	3.5 (2-7)	6 (2.75-8)
Satisfaction score, median (interquartile range)		49.5 (42.5-53.75)	53 (50-59)	52 (47.5-55)

^a^TECCU: Telemonitoring of Crohn’s Disease and Ulcerative Colitis.

^b^IBDQ-9: Inflammatory Bowel Disease Questionnaire 9.

^c^EQ-5D: EuroQol-5D.

^d^VAS: visual analog scale.

^e^eWPAI: Work Productivity and Activity Impairment.

### Health-Related Quality of Life

**Figure 6:**
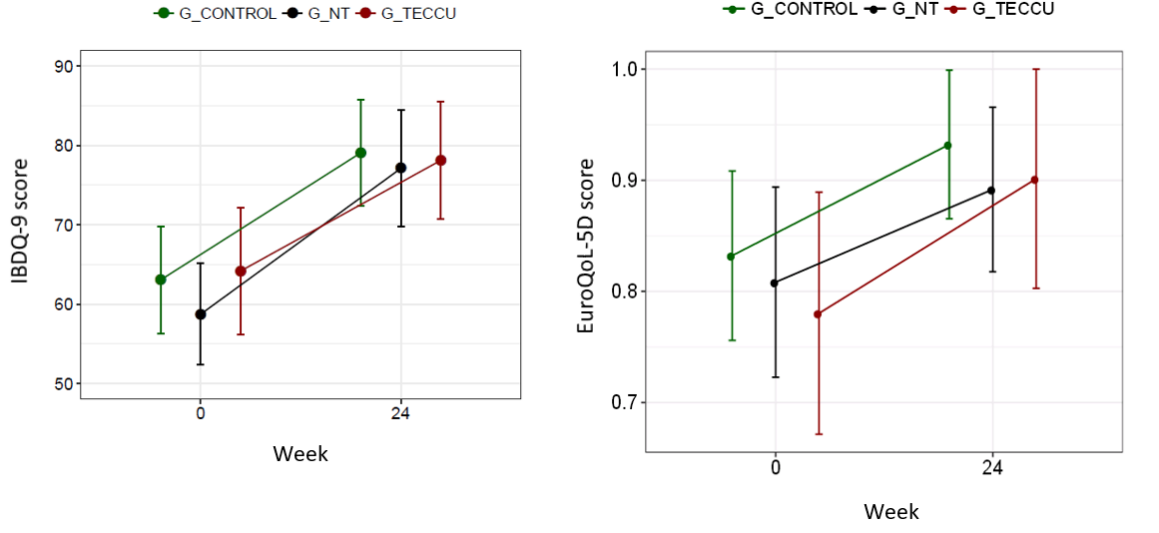
Evolution of the quality of life over the study period in the 3 groups. G_CONTROL: group receiving standard care with in-person visits; G_NT: group receiving nurse-assisted telephone care; G_TECCU: group receiving remote monitoring; IBDQ-9: Inflammatory Bowel Disease Questionnaire 9.

HRQoL scores were similar between all groups at baseline, and the HRQoL of patients from all 3 groups improved after 24 weeks. The median IBDQ-9 scores increased from 38.5 to 53 in G_control, from 37.5 to 53 in G_NT, and from 42 to 52.5 in G_TECCU (overall intervention effect on the IBDQ-9 score: OR=8.42, 95% CI=3.98-17.81, *P*<.001). The median EQ-5D score improved from 0.816 to 1.00 in G_control and from 0.825 to 1.00 in G_NT and G_TECCU (overall intervention effect on the EQ-5D score: OR=1.99, 95% CI=1.09-3.63, *P*<.001). However, the improvement in HRQoL was not significantly different among groups with regard to the IBDQ-9 score (G_TECCU vs G_control: OR=1.25, 95% CI=0.49-3.15, *P*=.64; G_NT vs G_control: OR=0.79, 95% CI=0.32-1.98, *P*=.62) and the EQ-5D score (G_TECCU vs G_control: OR=1.42, 95% CI=0.49-4.13, *P*=.52; G_NT vs G_control: OR=1.68, 95% CI=0.59-4.81, *P*=.33). In addition, a significant improvement in the HRQoL of patients from the 3 arms was evident in the EQ-5D-VAS scores, with an increase in the median score from 60.5% to 85% in G_control, from 62.5% to 70% in G_NT, and from 60% to 80% in G_TECCU (overall intervention effect on the score increase: OR=3.53, 95% CI=1.79-6.95, *P*<.001); however, the improvement was not significantly different between the groups at 24 weeks (G_TECCU vs G_control: OR=0.63, 95% CI=0.26-1.57, *P*=.32; G_NT vs G_control: OR=0.69, 95% CI=0.28-1.71, *P*=.43; Figure 6). The HRQoL improved in 4 patients in G_TECCU who were followed up via mobile phone, from a median baseline IBDQ-9 score of 31.5 to 47.75 and a median baseline EQ-5D score of 0.71 to 0.94, with an improvement in the median EQ-5D-VAS score from 50% to 70% at study completion.

### Work Productivity and Social Activities

With regard to work productivity and activity impairment, we compared the median percentage of work hours missed owing to disease on the basis of the answers to question 2 of the WPAI questionnaire (“During the past seven days, how many hours did you miss from work because of problems associated with your ulcerative colitis/Crohn’s disease?”). We found wide variability in the percentage of hours missed in the 3 arms, with no significant reduction in any group (OR=0.99, 95% CI=0.43-2.29, *P*=.93) and no differences among the groups at 24 weeks (G_NT vs G_control: OR=0.64, 95% CI=0.21-1.99, *P*=.44; G_TECCU vs G_control: OR=0.97, 95% CI=0.31-3.08, *P*=.96). Moreover, the median scores of impairment in work productivity over 12 weeks showed a larger reduction in G_TECCU than in G_control (OR=0.14, 95% CI=0.01-1.46, *P*=.10; G_NT vs G_control: OR=0.13, 95% CI=0.01-1.13, *P*=.06), but this improvement was not maintained at 24 weeks (G_TECCU vs G_control: OR=0.32, 95% CI=0.03-2.94, *P*=.31; G_AT vs G_control: OR=0.21, 95% CI=0.03-1.67, *P*=.14). With regard to social impairment, we found a significant improvement in daily activities and a significant reduction in the median score of each group at 12 weeks (OR=0.16, 95% CI=0.05-0.49, *P*=.002) and 24 weeks (OR=0.26, 95% CI=0.09-0.77, *P*=.02). This reduction in social impairment was larger in G_TECCU than in G_control at 24 weeks (OR=0.27, 95% CI=0.05-1.49, *P*=.13).

### Medication Adherence

**Figure 7:**
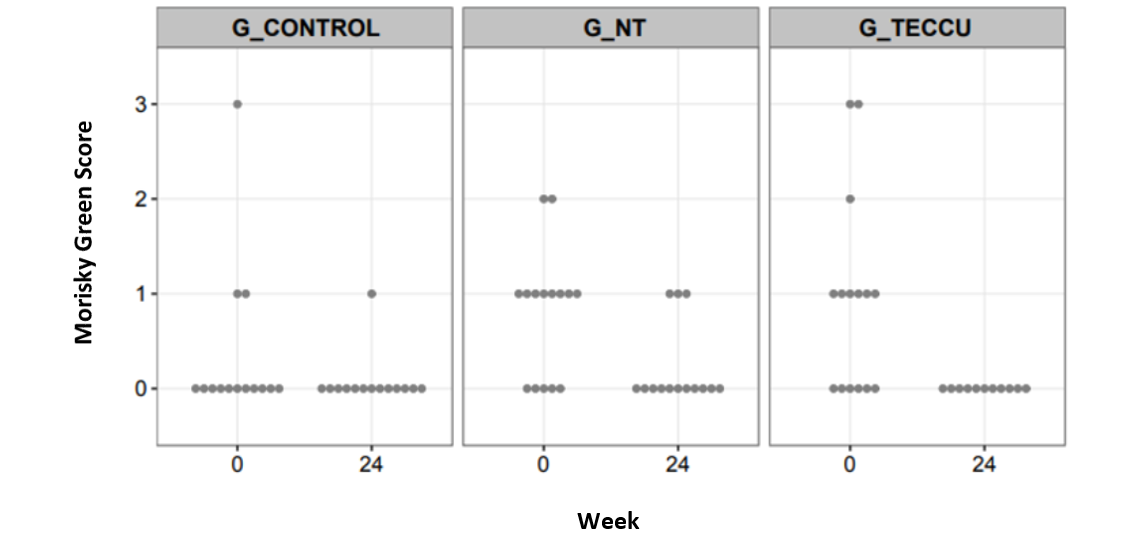
Evolution of the Morisky-Green score over the study period in the 3 groups. G_CONTROL: group receiving standard care with in-person visits; G_NT: group receiving nurse-assisted telephone care; G_TECCU: group receiving remote monitoring.

Medication adherence was poorer in G_NT than in the other 2 groups at baseline: 33.3% (7/21) of patients adhered to medication in G_NT; 66.7% (14/21), in G_control; and 57.1% (12/21), in G_TECCU. Despite these differences, medication adherence improved significantly in the 3 arms: 81% (17/21) in G_control, 71.4% (15/21) in G_NT, and 85.7% (18/21) in G_TECCU at 24 weeks (overall intervention effect on Morisky-Green score reduction: OR=0.051, 95% CI=0.001-0.769). In addition, all completers adhered to treatment in G_TECCU (Morisky-Green score=0), and the reduction in the Morisky-Green score was significantly more pronounced in G_TECCU than in G_control (OR=0.0001, 95% CI=1.02e^-10^ to 0.517; Figure 7).

### Safety Intervention

The safety of the 3 interventions was similar, and no differences were noted among the groups for visits to the emergency department (0 in G_TECCU, 2 in the G_NT, and 1 in the G_control), IBD-related surgeries (1 in each group), hospitalizations (2 in G_TECCU, 2 in G_NT, and 1 in G_control), and corticosteroid courses (4 in G_TECCU, 4 in G_NT, and 3 in G_control). Adverse effects of medication were reported in 8 patients (38.1%) in G_TECCU, 7 patients (33.3%) in G_NT, and 9 patients (42.9%) in G_control after 24 weeks (*P*=.92 for G_TECCU vs G_control). No patients died during the study, and adverse effects related to the follow-up intervention were not reported.

### Use of Health Care Resources

The total number of outpatient visits to the gastroenterologist or nurse after 24 weeks was lower in G_TECCU (72 visits; 25% of total) and G_NT (85 visits; 29.5% of total) than in G_control (131 visits; 45.5% of total). Similarly, the number of telephone calls was lower in G_TECCU (12 calls; 6.8% of total) than in G_NT (118 calls; 66.7% of total) and G_control (47 calls; 26.5% of total).

### Patient Satisfaction

Patient satisfaction improved from a median score of 52 to 57 in G_TECCU and from 49.5 to 55 in G_control (overall intervention effect: OR=8.93, 95% CI=2.97-26.84, *P*<.001) at 24 weeks; however, the satisfaction score remained unchanged at 53 points in G_NT. Satisfaction with previous care was high in the mobile phone subgroup of G_TECCU, with a median score of 54.25, although this score improved to 56.25 after 24 weeks of follow-up via mobile phone.

In G_TECCU, neither the patients nor the researchers perceived privacy breaches while using the Web platform. Only two patients in G_TECCU reported minor technical problems with temporary unavailability of the webpage over the follow-up period, but technical assistants resolved the issue remotely. The 3 patients who were noncompliant to the follow-up schedule were contacted by telephone, and 2 of them who were insufficiently compliant specified that they forgot to follow some remote controls because they were in acceptable health conditions. We subsequently scheduled in-person visits, but these patients only attended them once. The only patient who was lost to follow-up mentioned that neither the previous standard care nor the Web platform met her expectations; therefore, she decided to move to another center.

All staff, including 5 gastroenterologists and 2 nurses specializing in IBD, showed good acceptance of the platform and considered it easy to use. However, 2 physicians thought that the calendar included in the platform for health care providers to survey the next controls for each patient was not completely intuitive and would benefit from some change in its appearance. Furthermore, 1 physician reported minor technical problems with temporary unavailability of the webpage over the follow-up period, but the technical assistants resolved the issue remotely after 1 day.

## Discussion

### Principal Findings

We performed a randomized controlled clinical trial to compare the impact of a Web-based telemanagement system (TECCU), nurse-assisted telephone care, and standard face-to-face visits on health outcomes and outpatient visits in patients with complex IBD. Patients with IBD who start treatment with systemic corticosteroids, immunosuppressants, and biological agents for control of inflammatory activity are considered to have moderate-to-high complexity of disease. Our results showed that TECCU was safe and effective for improving health outcomes and the use of health care resources in this setting.

### Comparison With Prior Work

Although telemonitoring apps are well accepted and seem to be a safe approach for follow-up of patients in remission or with mild activity [6,18,41], the reported efficacy of telemonitoring on disease activity and QoL is inconsistent between studies [14,42], and no specific trials have evaluated the impact of telemedicine in patients with complex IBD. Therefore, we designed a controlled 3-arm clinical trial to assess the impact of the TECCU Web program on disease outcomes and health care use in comparison with the main strategies applied to date for the follow-up of patients with complex IBD (standard or telephone support provided by a nurse).

This program was designed in collaboration with patients and researchers to address the particular needs of patients with complex IBD, according to national and European clinical guidelines. As detailed elsewhere [27], this information was structured and filtered using an intelligent prioritization system, with generation of alerts and push notifications according to an integrated intervention protocol, which facilitated a rapid response from nurses and physicians for different events occurring during follow-up in a time period according to the severity of each alert. Furthermore, the Web platform allowed continuous communication between patients and health providers via electronic messaging, and we incorporated educational elements in the platform to improve disease knowledge among patients and empowerment through interactive materials. With the use of the TECCU Web platform, we found a significant improvement in HRQoL, medication adherence, social activities, and satisfaction; in addition, disease activity improved at the end of follow-up in each group. Moreover, a greater improvement in disease activity and social activities was reported with TECCU than with standard care, but the differences were not significant, probably due to the small sample size and the relatively short follow-up time.

In agreement with previous data [6,8], medication adherence was better in G_TECCU compared to the other groups and reached 100% among completers in G_TECCU, probably because care was continuously adapted at each stage of the disease and communication with physicians was better in this group. Therefore, we were able to easily identify eventual problems related to poor adherence [2]. In addition, constant tailored monitoring with Web TECCU could reduce interference with daily activities, as social impairment due to disease tended to improve in G_TECCU compared with the other two arms. Not surprisingly, the improvement in remission rates and social functioning was associated with high patient satisfaction and correlated directly with a significant improvement in HRQoL, measured by the specific IDBQ-9 and the EQ-5D.

The Web TECCU and nurse-assisted telephone care were associated with fewer outpatient visits than standard care, but the Web-monitoring system reduced the number of telephone calls compared with the other two groups. Our study was performed in a referral hospital with an accredited, well-structured IBD Unit and accessible outpatient clinics with specialized nurses and e-mail or telephone consultations. Therefore, the reduction of face-to-face visits suggests that the effect of the TECCU remote-monitoring system on the frequency of outpatient visits could be favorable in hospitals with referral IBD Units. Although this reduction in office visits could be influenced by the follow-up schedule used in our study, the frequency of visits for all the 3 arms was designed according to the standard clinical practice in our center and based on national and European guidelines. Furthermore, our Web-based telemanagement system was safe and well accepted by patients, with no adverse effects related to the study intervention and no differences among groups in terms of emergency visits, surgeries, hospitalizations, corticosteroid courses, or medication adverse effects, which is consistent with the findings of previous studies [6,8].

Despite different attrition rates in the use of telemedicine apps, our results indicate that it is important to emphasize the feasibility of telemedicine for providing solutions for the medical and social impact of IBD. In our study, only 3 patients did not complete the follow-up schedule in G_TECCU (1 patient was lost to follow-up and 2 patients were insufficiently compliant); this low proportion is likely a result of the reminder system integrated in the platform and the short follow-up period. However, previous clinical trials reported high withdrawal rates in telemedicine groups [6,8,18,22], despite adaptations in the platform design over recent years. Consequently, telemedicine is not suitable for all patients, and improvements in telemonitoring apps and the patient-selection criteria are necessary. Nevertheless, this is not necessarily a barrier to the application of telemedicine, because the majority of patients are adherent to remote monitoring, and the efficacy of these follow-up methods has been reported in different populations including patients with complex IBD, as reported in our trial. It is important to evaluate the disease phenotype, discuss preferences with the patient, and assess the ability to use ICTs. Some patients such as those in the immediate postoperative period and those with perianal disease, who require physical examination, may not benefit from remote monitoring with the technological tools available thus far.

### Strengths

The main strength of our study was its randomized controlled design, which allowed us to evaluate the impact of a Web telemanagement system on disease outcomes and compare the approach with the main strategies applied in the follow-up of IBD patients in daily practice. In addition, allocation concealment ensured that researchers were blinded to group assignment during the randomization process. Furthermore, the selection of patients with complex IBD from a referral center provides important data on the effect of telemedicine in patients with moderate-to-severe disease activity or initiating administration of immunosuppressants and biological agents, which is a specific population that has not been sufficiently represented in previous studies. Another advantage was the selection of the activity index and biological tools for measuring disease activity and other outcomes remotely. The Walmsley index was recently validated for self-administration via an online tool [43] and correlates well with other, more complex-activity indexes based on endoscopy [44]. Similarly, the reduced 6- and 9-point versions of the Mayo index, which are not based on endoscopy, show clinical response to treatment, similar to the full Mayo score [30]. Finally, as HBI may not completely reflect the inflammatory activity of the disease [45], the use of biological markers (C-reactive protein and FC) provided added value to remote monitoring, because they are sufficiently sensitive for detecting mucosal inflammation [46], and FC levels can be easily recorded at home.

### Limitations

Our study was subject to a series of limitations. First, considering the specific study population, the sample was not large enough to detect statistically significant differences in clinical activity and the types of interventions assessed; as such, neither the patients nor the researchers were masked to the intervention. It is possible that the differences estimated among groups in the scarce literature on the impact on health outcomes could interfere with the sample size calculation in this pilot trial, and the lack of blinding could improve the usual attention provided to patients in G_control, which could also attenuate differences in these outcomes among groups. Nonetheless, our results were analyzed by an independent statistician who was blinded to group identification. Second, the selection of patients with complex IBD in a tertiary referral hospital prevented extrapolation of our results to the entire IBD population. Third, we did not perform colonoscopy in all cases, because it increases costs and risks to patients and is not performed routinely in all patients during daily practice. Instead, we used biological markers that were highly sensitive for endoscopic activity. Finally, the 24-week follow-up period could be considered a short duration, because both patients and physicians need time to learn how to interact with the platform, thus leading to interference with the efficacy of the Web system in terms of disease outcome in the experimental group. Therefore, trials with longer follow-up periods are required to confirm the efficacy of telemedicine in improving long-term disease outcomes.

### Conclusions

TECCU is a safe tool for the control of disease activity and improves HRQoL, medication adherence, and impairment in social activities in patients with complex IBD. In addition, it reduces the use of health care resources and the number of outpatient visits and telephone calls. It is important to select patients who are willing and able to use Web programs, since the results of this study suggest greater efficacy in health outcomes with a consequent improvement in the HRQoL and satisfaction of patients who adhere to telemanagement. Web-based systems are a safe and feasible option for IBD monitoring and could play a key role in reorganizing the structure of health systems if they prove to be cost-effective in the long-term. On the other hand, although telemonitoring platforms are well accepted, thus far, they have not clearly demonstrated their efficacy on health outcomes in patients with complex IBD and those in remission or with mild-to-moderate disease. Future studies with longer follow-up periods are necessary to confirm our findings in order to determine whether Web-based programs can improve the long-term course of IBD in a more-complex setting.

## Appendix

Multimedia Appendix 1 CONSORT-EHEALTH checklist (V 1.6.1).

## Abbreviations

CD: Crohn disease
EQ-5D: EuroQol-5D
FC: fecal calprotectin
G_control: group receiving standard care with in-person visits
G_NT: group receiving nurse-assisted telephone care
G_TECCU: group receiving remote monitoring
HBI: Harvey-Bradshaw index
HRQoL: health-related quality of life
IBD: inflammatory bowel disease
IBDQ-9: Inflammatory Bowel Disease Questionnaire 9
ICT: information and communication technologies
SCCAI: Simple Clinical Colitis Activity Index
TECCU: Telemonitoring of Crohn’s Disease and Ulcerative Colitis
UC: ulcerative colitis
VAS: visual analog scale
WPAI: Work Productivity and Activity Impairment
